# Mitigating Transmission Errors: A Forward Error Correction-Based Framework for Enhancing Objective Video Quality

**DOI:** 10.3390/s25113503

**Published:** 2025-06-01

**Authors:** Muhammad Babar Imtiaz, Rabia Kamran

**Affiliations:** 1 Software Research Institute, Technological University of the Shannon: Midlands Midwest, N37 HD68 Athlone, Ireland; 2 Department of Information Security, The Islamia University of Bahawalpur, Bahawalpur 63100, Pakistan

**Keywords:** error correcting code (ECC), advanced video coding (AVC), confidentiality, selective encryption, secure multimedia transmission, error mitigation, error detection, quality of experience (QoE), video transmission system, error recovery, perceptual video quality

## Abstract

In video transmission, maintaining high visual quality under variable network conditions, including bandwidth and efficiency, is essential for optimal viewer experience. Channel errors or malicious attacks during transmission can cause degradation in video quality, affecting its secure transmission and putting its confidentiality and integrity at risk. This paper presents a novel approach to enhancing objective video quality by integrating an energy-efficient forward error correction (FEC) technique into video encoding and transmission processes. Moreover, it ensures that the video contents remain secure and unintelligible to unauthorized parties. This is achieved by combining H.264/AVC syntax-based encryption and decryption algorithms with error correction during the video coding process to provide end-to-end confidentiality. Unlike traditional error correction strategies, our approach dynamically adjusts redundancy levels based on real-time network conditions, optimizing bandwidth utilization without compromising quality. The proposed framework is evaluated across full reference objective video quality metrics, demonstrating significant improvements in the peak signal-to-noise ratio (PSNR) and PSNR_611_ of the recovered videos. Experiments are carried out on multiple test video sequences with different video resolutions having various characteristics, i.e., colors, motions, and structures, and confirm that the FEC-based solution effectively detects and corrects packet loss and transmission errors without the need for retransmission, reducing the impact of channel noise and accidental disruptions on visual quality in challenging network environments. This study contributes to the development of resilient video transmission systems with reduced computational complexity of the codec and provides insights into the role of FEC in addressing quality degradation in modern multimedia applications where low latency is crucial.

## 1. Introduction

The transmission of digital videos over error-prone channels corrupts the bitstream, causing an unpleasant effect on objective video quality. Every time a video is delivered to an end user, it is first passed through various processing platforms to get encoded, compressed, digitized, quantized, decompressed, decoded, and transmitted through various communication channels. The quality of the video is a measure of the amount of deterioration in the video caused by any processing or transmission system when compared to the original video. It determines the extent to which the video has changed its originality after going through all of these processes. The appearance and motion of objects seem smooth in high-quality videos. Errors during transmission cause degradation in the smoothness, resulting in lower video quality.

There are three fundamental security services that can be compromised during the communication of data over a network due to malicious attacks and transmission errors. *Confidentiality* ensures the privacy and secrecy of data and guards against illegal access to data. *Integrity* refers to the completeness of transmitted data and guarantees that the authorized users have been given all of the relevant information. *Availability* ensures that the required data must be provided to the user at the requested time. When a video is transmitted over an error-prone network, its integrity may be affected, as the received video might be altered due the encountered errors, resulting in modified video contents. The proposed method provides an efficient technique for the detection and recovery of such errors to compensate the originality of the video. Furthermore, in advanced multimedia systems, ensuring data confidentiality and reliability has become increasingly crucial. As devices evolve to be faster and resource-constrained, it is vital to enhance their resistance to attacks without introducing additional hardware complexity or increasing the computational cost. Thus, our proposed framework ensures the confidentiality and integrity of the video against security threats and errors by merging encryption and decryption with error correction within the video coding process, reducing the complexity of joint schemes to enable efficient implementation.

Video quality can be measured either subjectively or objectively. *Subjective video quality* is the assessment of video quality from the end user’s perspective. It is performed by asking a particular user their opinion about the quality of the video at the receiving end after passing through all of the processing stages. Subjective video quality assessment is highly dependent on the observer, the environment in which the observation is conducted, and the elements that are considered to deduce the results by the observer (user preferences in terms of color, brightness, display size, or resolution). *Objective video quality* is the measure of quality degradation of the video as it goes through a number of processes, including encoding, compression, and transmission. Calculating the mean square error (MSE), signal-to-noise ratio (SNR), peak signal-to-noise ratio (PSNR), structural similarity index (SSIM), and video multimethod assessment fusion (VMAF) is a commonly used mathematical method to predict video quality [1,2]. Objective video quality evaluation techniques can be categorized as full reference (FR), reduced reference (RR), or no reference (NR) depending on the amount of information from the original video being used for comparison [3]. FR techniques compare each and every pixel of the original and impaired videos, unaware of the processes applied in between. RR procedures use some of the characteristics from both of the videos for comparison. NR methods attempt to recover the impaired video without knowing anything about the original video [4,5,6].

**Figure 1:**
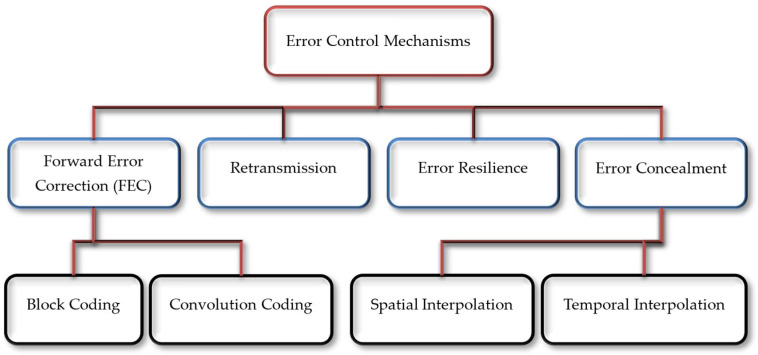
Error control mechanisms for video coding.

Error correcting codes (ECC) are used while encoding the data before transmission to identify any change that may occur during transmission. ECC for video applications can be categorized into four different mechanisms: forward error correction (FEC), retransmission, error resilience (ER), and error concealment (EC) [7]. A hierarchical representation of error controlling schemes is shown in Figure 1. FEC techniques add redundant bits to the original bitstream for error recovery. FEC can be carried out in two ways; block coding and convolution coding. Block codes work on the data in fixed-sized blocks, whereas convolution codes work on bitstreams of arbitrary size. In retransmission techniques, the receiving device sends an acknowledged signal to the transmitting device to acknowledge the received or lost packet and requests the sender to retransmit the data. It is applicable only when the number of errors is small, e.g., the automatic repeat request (ARQ) needs a backchannel between two communicating devices and takes additional time to retransmit the data, which makes it unsuitable for interactive real-time video transmission, broadcast, unicast, and multicast applications [8,9]. ER methods are used to prevent error propagation in a bitstream during transmission. EC provides methods for hiding the effects of errors or packet loss and presents the visual information in such a way that makes those errors unnoticeable by the user [10]. Spatial interpolation regenerates the missing data in intra-coded frames using neighboring pixels, while temporal interpolation is used to reconstruct lost data in inter-coded frames using other reference frames [11,12].

**Figure 2:**
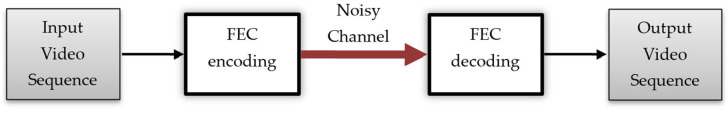
Basic mechanism of forward error correction in video coding.

An FEC technique is used in our work to recover errors that occur during transmission to avoid the need to retransmit the data. These errors may affect the quality, visibility, and completeness of information by causing blurriness, color alteration, false edges, jagged motion, flickering, and chrominance in the video sequence [13]. It makes broadcasting, video streaming, and real-time applications more efficient. FEC is generally used for the transmission of video, audio, and other signals for which a transmission backchannel is not available and retransmission is not possible in case of error occurrence. Moreover, the huge size of video files will take a lot more time if retransmitted. So, FEC provides a better and time-saving mechanism to cover up the damage caused by these errors [14]. Redundant bits are added to the data before transmission during the encoding process, which are used to indicate an error and provide a mechanism to find the location of the error for recovery while decoding the incoming data [15]. Figure 2 presents the general scheme we have adopted using the H.264/AVC video coding standard for FEC to reduce the effect of channel errors on objective video quality.

Secure transmission of multimedia content is crucial, as encrypting the entire video bitstream using encryption standards like the Advanced Encryption Standard (AES) is computationally complex, increasing latency and energy consumption due to pre-processing and post-processing operations. Selective encryption has emerged as an alternative that involves encrypting only the most sensitive and confidential contents of the video in compressed domain. This technique enables format compliance and preserves coding efficiency, providing a low-complexity solution for video transmission systems [16,17]. The integration of selective encryption with error correction within the video coding process aims at enhancing the overall robustness and efficiency of the proposed scheme. This joint approach provides enhanced security and improved reliability, as selective encryption ensures protection against access attacks, reducing the computational overhead while maintaining confidentiality, whereas FEC detects and corrects errors during transmission, ensuring video quality and integrity. This integrated framework reduces the overall complexity and computational requirements, and it protects against both security threats and transmission errors, including packet loss, data corruption, and unauthorized access. It ensures a higher quality of service (QoS), providing a better viewing experience for end users.

A variety of video codecs are used to implement compression techniques on huge-sized video to reduce the size during the encoding process before transmission. These codecs operate at the application layer (Layer 7) of the OSI model, which is responsible for providing services and interfaces so the application can communicate properly. Error correction at the application layer provides better support for real-time applications by maintaining a high quality of experience for users by minimizing the impact of transmission errors. In our work, the H.264/AVC codec encodes the video and reduces its size to accommodate the issues of limited bandwidth and storage capacity of the transmission channel [18]. Selective encryption is preferred to encrypt only the selected elements of the video due to its large size, as this method facilitates processing and computational requirements while protecting the contents. Extra parity bits added by the FEC algorithm take more bandwidth than the actual data, but the video files are generally large in size, particularly the latest trend of FHD, UDH, and 4K videos, and require more time to retransmit in the case of any data loss or error. Thus, to compensate the quality of the video on the receiving side, an error correction mechanism is introduced in this work for reliable delivery of data.

The existing methods for error correction demand complex computations and require additional storage capacity and complex prediction methods. This method provides better error recovery with minimum computational cost and improved performance, reducing the effect of accidental disruptions on the perceptual quality of the video. It targets the challenges of ensuring reliable video transmission at the application layer, where the unique characteristics of video data and maintaining QoS necessitate FEC strategies for improved end-to-end reliability. The problem we aim to address is the degradation of video quality due to transmission errors that are not fully mitigated by the error correction mechanisms at lower layers.

The following research contributions are achieved through the proposed framework:

1: *Optimization of FEC Framework for Seamless Video Transmission System*: This study proposes an FEC mechanism that robustly adjusts redundancy levels in response to real-time network conditions. This method minimizes the impact of packet loss and channel noise while optimizing bandwidth usage without requiring retransmissions.
2: *Improvement of Objective Video Quality Metrics*: The proposed framework achieves measurable enhancements in PSNR and PSNR_611_ by integrating the proposed FEC framework with H.264/AVC video coding. The framework effectively addresses the challenges posed by variable video characteristics, such as motion, structure, and resolution.
3: *Integration of Selective Encryption and Error Correction within the Video Coding Process*: The proposed technique combines the FEC mechanism with H.264/AVC syntax-based selective encryption and decryption algorithms using specific syntax elements of the video to ensure confidential and reliable video transmission system.
4: *Adaptiveness to Various Video Resolutions*: This method is not limited to videos with a specific resolution and can be deployed on multiple video resolutions. Its working efficiency is tested on video sequences of CIF and HD resolution in our work and the results prove its adaptiveness to the resolution flexibility of videos.
5: *Effectiveness on Different Quality Perception Values on Video Quality*: The proposed framework is implemented at different QP values to assess the effect on video quality. It shows that perception quality is inversely related to video quality.

The rest of this paper is structured as follows. Section 2 reviews the related work on this topic. Section 3 describes the materials and methods of the proposed framework. Section 4 illustrates the results of the proposed framework on different test video sequences. Section 5 provides a discussion and suggestions related to the outcomes of this work. Section 6 concludes the paper by providing an overview of this work and its adaptiveness for quality enhancement due to error-prone channels in modern communication.

## 2. Related Work

A variety of solutions have been explored in the literature to mitigate video quality concerns over wireless networks. The integration of FEC techniques enables the receiver to detect and correct errors in real-time applications by embedding redundant bits into packets during transmission. It mitigates delay impacts, ensuring industrial wireless systems meet stringent latency and reliability requirements [19]. Chen et al. [15] proposed a novel RL-AFEC approach based on frame-level Reed–Solomon (RS) codes that can learn to optimize FEC paraments in real time to minimize latency and packet loss by automatically adjusting the redundancy rate for each frame. In [17], the authors provide a survey on crypto-coding, joint encryption, and error correction techniques to simultaneously ensure the confidentiality and reliability of data transmission. They classify the joint schemes into three categories: joint encryption and channel coding (JECC), joint encryption and source coding (JESC), and joint encryption and network coding (JENC). JECC includes code-based encryption, lattice-based encryption, and hash-based encryption; JESC includes compressive sensing-based encryption and transform domain encryption; and JENC encompasses network coding-based encryption and secure network coding. Our proposed work is based on JESC transform domain encryption, which combines encryption and source coding to compress and encrypt data simultaneously using the H.264/AVC video codec.

Bagheri et al. [20] proposed a novel joint encryption, channel coding, and modulation scheme, called the quasi-cyclic low-density parity check (QC-LDPC) lattice code, to provide improved security, better error correction, and efficient transmission over wireless networks. Their approach implements lattice-based encryption to offer semantic security, QC-LDPC codes for robust error correction, and a lattice-based modulation scheme for robust transmission. However, it requires complex computations and secure key management, which we have focused on to optimize in our work. A novel method that adapts to changing channel conditions using deep learning was designed in [21]. This adaptive FEC approach was based on long short-term memory (LSTM) neural networks, employing frame-level RS coding to dynamically select appropriate redundancy levels to achieve higher VMAF scores, but it requires significant computational resources and a large training dataset to successfully implement, which may be difficult to obtain.

A combination of subjective and objective video quality assessments was conducted in [22]. For subjective quality assessment, a user study with 40 participants was conducted, whereas various FR and NR quality metrics, including PSNR, SSIM, MS-SSIM, ST-RRED, FAST, and VMAF, were used for objective video quality assessment. The authors suggested the use of the H.264 codec over newer codecs such as HEVC, VP9, and AV1, as 91% of video streaming services utilize it and most browsers and devices do not provide full support for advanced standards. The joint-channel rate distortion (RD) optimization method presented in [23] minimizes end-to-end distortion of video signals and optimizes bitrate allocation among different video frames using a combination of RS and convolutional codes. Although it protects video signals against channel errors, it is not suitable for scalable high-resolution videos and involves complex computations. In [24,25], the authors evaluated the performance of various video coding standards, including H.264/AVC, H.265, AV1, VP9, HEVC, and VVC, to check the similarity between original and compressed videos using a machine learning-based approach to predict optimal encoding paraments such as video sequence, resolution, and bitrate. Their work discussed several quality assessment metrics, such as PSNR_611_, SSIM, and VMAF, and suggested the use of PSNR_611_ objective video quality metrics over other methods. Context-adaptive binary arithmetic coding (CABAC) and context-adaptive variable-length coding (CAVLC), two H.264 codec entropy coders, were discussed to examine the comparative effect of channel errors on selectively encrypted videos in [26]. They concluded that CAVLC was more susceptible to channel errors than CABAC in analyzing the combined effect of selective encryption and compression on video quality. Since all advanced and hybrid encoders support CABAC, this entropy encoder was selected for developing the proposed error correction technique in our work.

A selective encryption method for H.264/AVC videos based on CABAC is presented in [27], involving zig-zag scanning followed by encryption of discrete cosine transform (DCT) coefficients, which significantly impacts the texture and content of videos during compression. The scrambling process efficiently provides confidentiality by rearranging macroblocks (MB) of data in a way that makes unauthorized access difficult. An H.264/AVC syntax-based selective encryption method utilizing multiple syntax elements, such as residual coefficients (RCs), transform coefficients (TCs), and motion vectors (MVs), to scramble video contents is presented in [28]. Another selective encryption scheme using the CABAC encoder for VVC is proposed, which identifies TCs, MVs, intra-prediction, and inter-prediction modes as sensitive syntax elements. This scheme encodes syntax elements and selects encoded bins for encryption, which are then encrypted using a symmetric-key encryption algorithm [16]. Selective encryption schemes can be categorized as syntax element-based, bitstream-based, and hybrid encryption. Syntax element-based encryption encrypts specific syntax elements; bitstream-based approaches encrypt the entire bitstream, including syntax and non-syntax elements; whereas hybrid approaches combine both of them to provide robust security. An H.265/HEVC-based hybrid scheme of symmetric-key selective encryption was proposed using the CABAC encoder, which targets sensitive syntax elements including the transform unit (TU), motion vector differences (MVDs), intra-prediction modes (IPMs), inter-prediction modes (InterPMs), and coding unit (CU) flags. The quality of encrypted frames is evaluated through PSNR and SSIM [29]. In [30], the authors proposed AES-CTR encryption built upon an improved CABAC algorithm to selectively encrypt syntax elements, including the suffixes of sign bits, absolute values of residuals of MVDs, DCT coefficients, and QPs. Their proposed approach encrypts specific parts of the video, ensuring sufficient protection from unauthorized access while maintaining format compliance, thus achieving a balance between security and efficiency, making it suitable for real-time applications. Similarly, a 4D hyperchaotic algorithm using the CFB mode of AES is implemented in [31] for privacy protection of videos based on different syntax elements (IPM, MVD, residual coefficients, and delta QPs). PSNR and SSIM reference indicators are used to measure the perceived effect of the video.

A selective encryption scheme for H.264/AVC video content is proposed in [32], encrypting only critical parts such as IPMs, MVDs, and RCs. It balances security and efficiency and ensures format compliance utilizing the chaos-based approach to offer reduced computational overhead. However, its security is dependent on the chaotic system and requires careful consideration of key management and security threats. In [33], the authors review existing selective encryption schemes in HEVC and suggest a selective encryption method that generates encrypted bitstreams, records syntax elements, and reconstructs original elements to design a pseudo-key stream for decryption. Experimental analysis is conducted across various QPs to assess the effectiveness of the scheme. The authors in [34] present a secure and efficient data hiding method in encrypted H.264/AVC bitstreams, using IPMs and MVDs to protect information related to texture and motion. An additional security layer is achieved through RCs. However, data hiding may lead to potential video quality degradation and additional computational complexity. Several schemes proposed in [35] encrypt video content by scrambling the IPMs of intra-coded macroblocks. Exclusive OR (XOR) is used to offer data security. The authors of [36] provide a review of existing and encryption techniques for H.264/AVC video encoders, which are categorized on the basis of the stages where the encryption is applied: before compression, through compression, and after compression. These encryption techniques are based on IPMs, residual data, MVDs based on XOR operation, transformation, and the entropy coding process.

The existing literature suggests numerous schemes of error recovery for enhancing video quality that involve complex mathematical computations and result in high computational cost. The method proposed in this work involves a bit-inversion mechanism that detects the error bit from the bitstream and flips it to recover the error and improve video quality at the receiving end. The proposed technique additionally merges the error recovery process with syntax-based encryption to preserve the confidentiality and integrity of videos for improved user experience.

## 3. Materials and Methods

In this study, we have incorporated the novel approach of forward error correction while crypto-encoding videos before transmission using the H.264/AVC encoder to recover the errors encountered during transmission, resulting in improved objective video quality. Additionally, the proposed work enhances transmission security by selectively encrypting videos using specific syntax elements to protect against unauthorized access. The details are described in subsequent subsections.

### 3.1. Encoding and H.264/AVC Syntax-Based Selective Encryption

Due to the huge size of videos, they must be compressed to reduce their size to accommodate bandwidth limitations. Video is a series of frames that rapidly change over a given time to illustrate moving objects. Video codecs arrange these video frames in groups, known as a group of pictures (GOP), which are compressed and encoded as independent sets of video frames. Each frame is composed of a set of slices, which are composed of macroblocks. These macroblocks are further divided into a number of blocks. Each macroblock is a 16 × 16 array of pixels. A block is represented by a matrix of 4 × 4 pixels, which is the smallest unit of compression system. The H.264 video codec has two forms of entropy coders, both of which perform lossless compression: context-adaptive binary arithmetic coding (CABAC) and context-adaptive variable-length coding (CAVLC). Both of these forms are context adaptive, which means that compression is dependent on the patterns of the coefficients that are extracted from the coefficient matrix formed after transform coding and quantization. As both of these entropy coders perform lossless coding, the resultant bitstream is very close to the original input stream. The purpose of this study was error recovery to improve the visual quality of the received videos that has been compromised during transmission. CAVLC is more sensitive to errors, whereas CABAC is not much affected by channel errors, which makes it a better choice for implementing the proposed FEC algorithm [26].

There are multiple residual parameters of the H.264/AVC CABAC entropy coder, including TCs, MVDs, delta QPs, and the arithmetic signs of TCs and MVDs. We have used the sign bits of TCs and MVDs for XOR-based selective encryption at the final step of the encoding process in our suggested framework to preserve format compliance and security during transmission. The video frame is divided into 4 × 4 blocks. The bits within a 4 × 4 block are first shuffled and the TCs and MVDs are selected for applying XOR encryption on the encoded bitstream of the shuffled blocks using a 128-bit encryption key. Let $C_{MVD}$, $C_{TC}$, and *k* represent the ciphered MVDs, ciphered TCs, and the key, respectively, then the ciphertext is generated through the bitwise XOR encryption process *E*, as follows:

(1) $$C_{MVD}∶=E(k,\;MVD)=k⊕MVD$$

(2) $$C_{TC}∶=E(k,\;TC)=k⊕TC$$

The process is reversed on the receiving end by the decryption algorithm *D*, where the symmetric key is XORed with the ciphered MVDs and TCs of each block after extraction of the encrypted MVDs and TCs to reconstruct the original bitstream by combining the decrypted blocks, as follows:

(3) $$M_{MVD}∶=D(k,\;C_{MVD})=k⊕C_{MVD}$$

(4) $$M_{TC}∶=D(k,\;C_{TC})=k⊕C_{TC}$$

where $M_{MVD}$ and $M_{TC}$ are the original MVDs and TCs after decryption used to reconstruct the original block.

### 3.2. Redundancy

The most basic concept in error control mechanisms is redundancy. When the digital data are transmitted in the network, a few redundant bits are added to the original data during the encoding process. These redundant bits are used to detect and correct the errors that have occurred during transmission. These additional bits are added on the sending side, which are then removed at the receiving side after the transmission is complete. Redundant bits are also known as *parity bits* or *check bits*. Parity bits add checksums in the data that enable the receiving device to check the occurrence of errors. A parity check counts the number of 1s in the dataword. There are two methods to add redundant bits on the basis of a parity check: even parity and odd parity. In *even parity*, the parity bit is assigned a value of 1 if the number of bits having 1s is odd; if the number of bits having 1s is even, then 0 is assigned to the parity bit. So the total number of 1s will be even to maintain even parity. In *odd parity*, if the number of 1s is even, the parity bit is set to 1 to make it odd; if the number of 1s is odd, 0 is assigned to make odd parity.

### 3.3. Block Coding

**Figure 3:**
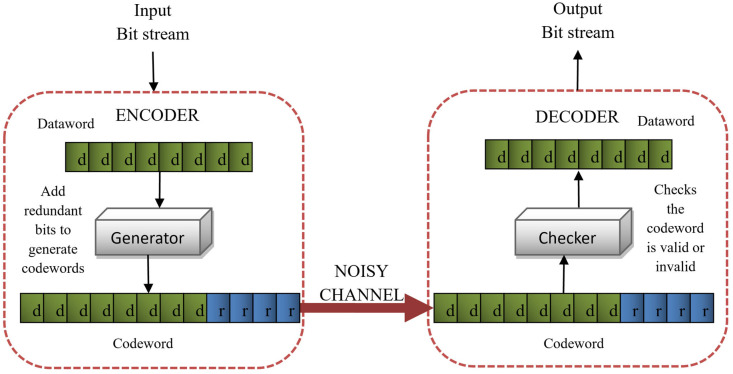
Error detection process in block coding.

In our work, we used a block coding FEC method, which works on the macroblock of a video frame. In *block coding*, the message is divided into blocks of fixed size called *datawords*. A few redundant bits are added to each dataword to generate *codewords*. Redundancies can be added by using different scenarios. It can either be added to the start of the message, at the end of the message, or somewhere in between [19]. Figure 3 shows the basic mechanism of error detection in block coding.

Let *d* be the number of bits in a dataword and *r* be the number of added redundant bits, the resultant block of *c*-bits is called a codeword, where *c* = *d* + *r*. *d*-bits can represent *2^d^* different datawords. Similarly, *c*-bits can represent *2^c^* different combinations of codewords. Since *c* > *d*, the number of codewords would be greater than the number of datawords. For each dataword, there exists only one codeword, so there will be *2^c^* − *2^d^* extra codewords that are considered as invalid. A codeword is accepted on the receiving end only if it is a valid codeword, or else it is discarded by the decoder.

### 3.4. The Gilbert–Elliott Channel Model

We implemented the Gilbert–Elliott channel model in our work to model the noisy communication channel [37]. This model is computationally efficient and produces an error burst to demonstrate the accurate effect of errors on an application without involving the physical processes [38,39,40]. These errors may result in loss or alteration in packets, frames, or bits from the transmitted bitstreams [41,42].

The Gilbert–Elliot (GE) channel model is a discrete time hidden Markov chain model that consists of two states, i.e., the good state and the bad state. Suppose that *S* = {*G*, *B*} is the state space of the wireless communication channel, where *G* and *B* are the good and the bad states, respectively. The probability of error occurrence in good state *G*, denoted by *P*(*G*), is relatively lower than the probability of error occurrence in bad state *B*, denoted by *P*(*B*). When the error occurrence in the good state does not happen, then *P*(*G*) will be 0, indicating the error-free transmission of bits through the channel, and *P*(*B*) will become 1, indicating the occurrence of error in the bad state. In order to generate fewer erroneous bits, it is assumed that *P*(*G*) > *P*(*B*), i.e., the good state is more likely to experience error bits than the bad state.

**Figure 4:**
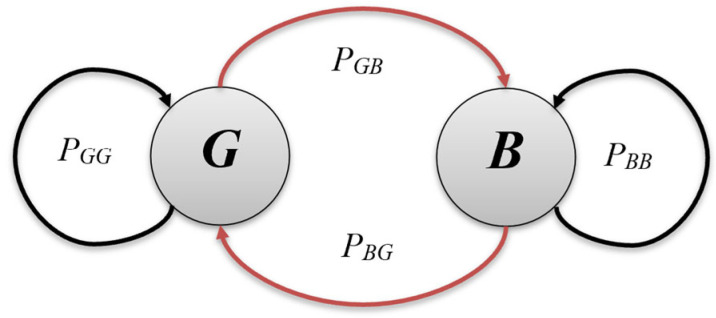
Gilbert–Elliott channel model.

The probability of shifting from one state to the other state is known as *transition probability.* Let *P_GG_* be the probability that the next state is again a good state and *P_BB_* be the probability that the next state is again a bad state. *P_GG_* and *P_BB_* are known as self-transition probabilities. Similarly, *P_GB_* and *P_BG_* denote cross-transition probabilities, i.e., *P_GB_* is the probability that the next state is bad state *B* from the current state *G*, whereas *P_BG_* is the probability that the next state is a good state considering the present state *B*. A state transition diagram of the GE channel model is given in Figure 4.

The Gilbert–Elliot Model uses a two-state transition matrix of order two-by-two to determine the state transition probabilities. The two-state transition matrix determines the state transition probabilities and is represented by M in (5), as follows:

(5) $$M=\left[\begin{array}{cc} P_{GG} & P_{GB}\\ P_{BG} & P_{BB} \end{array}\right],$$

The sum of all probabilities from a particular state is always 1. Thus, from the state-transition diagram shown in Figure 4, it is concluded that:

(6) $$P_{BB}+P_{BG}=1\;and\;P_{GG}+P_{GB}=1$$

From (6), the self-transition probabilities (7) and cross-transition probabilities (8) can be calculated as follows:

(7) $$P_{BB}=1-P_{BG}\;and\;P_{GG}=1-P_{GB}$$

(8) $$P_{BG}=1-P_{BB}\;and\;P_{GB}=1-P_{GG}$$

The expected amount of time for which the channel remains in one state before moving to the other state (either in *G* or *B*) is known as the mean sojourn time of being in that state. There were only two states in our GE channel model; therefore, the mean sojourn times of good state *G* and bad state *B* are denoted by *T_G_* and *T_B_*, respectively. The mean state sojourn times *T_G_* of state *G* and *T_B_* of state *B* can be estimated by Equation (9), as follows:

(9) $$T_{G}=\frac{1}{1-P_{GG}}\;and\;T_{B}=\frac{1}{1-P_{BB}}$$

The probability that the channel errors occur in steady state is known as the steady state probabilities, denoted by *P_GG_* and *P_BB_* depending on the steady state being *G* or *B*, respectively. Steady state means that the state of the channel remains unchanged. The probability of being in steady state *G* (*P_GG_*) and the probability of being in steady state *B* (*P_BB_*) are dependent on their mean sojourn times and are computed using (10):

(10) $$P_{GG}=\frac{T_{G}}{T_{G}+T_{B}}\;and\;P_{BB}=\frac{T_{B}}{T_{G}+T_{B}}$$

The number of bit errors per unit time is known as the mean bit error rate (BER) (M_BER_) and can be obtained by using the following equation:

(11) $$M_{BER}=\left(P_{GG}\times P(G)\right)+(P_{BB}\times P(B))$$

It is assumed that *G* state is error-free, meaning that *P*(*G*) = 0. All of the bits of the frame are likely to be transmitted correctly in the good state. The good state is considered error-free because it represents a period of time when the channel is in favorable condition with low error probability. This assumption allows the model to show a burst nature of the transmission channel by focusing on capturing the characteristics of the bad state where errors are more likely to occur. This simplification provides a reasonable approximation for communication system analysis [43]. The probability of being in a good or bad state is dependent on *M_BER_* [44]. The GE model is used to introduce bit errors in transmitted bitstreams, assuming that the occurrence of bit errors is independent of each other. It calculates the steady state probability, transition probability, and bit error rate at each state. In our study, all of the bits in the frames that are in state *G* are supposed to be transmitted without any error. This reduces the computational cost of the traditional GE model.

### 3.5. Error Detection and Correction

We proposed a block coding mechanism for error control that detects the single bit error in each block of transmitted data. Our aim was to maximize error recovery by dividing the frame into blocks of specific length and adding redundancies, as shown in Figure 3. The *generating function* in the encoder generates the codewords by adding parity bits to each macroblock of the video frame. The incoming bitstream is then compared to the list of valid codewords, which is already being sent to the decoder for detecting errors. The *checking function* in the decoder performs this comparison. If the received codewords match the valid codewords, it means the data are error-free. Otherwise, an error has occurred that altered the bits during the transmission. Error detection simply indicates the presence of errors without revealing their quantity or location. Error correction is a bit complex, as it requires knowledge of both magnitude and location of errors. To correct any error, it is essential to know whether the data are transmitted error-free or are corrupted during transmission. Therefore, error detection precedes error correction, serving as a crucial step in ensuring data integrity.

### 3.6. Proposed Forward Error Correction Framework

Forward error correction is an error correcting technique in which the data are recovered from errors that occur during transmission. Unlike ARQ (automatic repeat request), when an error is detected, FEC restores the data that are affected by the channel errors without requesting retransmission from the sender. In FEC, redundant bits are added to the original bitstream by the FEC encoder. The decoder uses these redundant or additional bits to guess the original data in case the original bits are corrupted or lost during transmission. We inserted errors into YUV video sequences by implementing the GE channel error model with the help of the H.264/AVC CABAC entropy coder to test the working of our proposed FEC method, which can detect up to one error in each macroblock of the video frame. Each macroblock is a 16 × 16 array of pixels.

The proposed framework involves the following steps implemented, as shown in Algorithm 1, using the H.264/AVC CABAC entropy encoder to encrypt and then correctly guess and correct the errors that occurred due to a noisy channel to provide recovered data to the receiver. The H.264/AVC CABAC entropy coder was used via the JSVM software tool, developed by the Joint Video Team (JVT) of ITU-T and ISO/IEC, with contributions from Fraunhofer HHI, Berlin, Germany.
**Algorithm 1** Overall steps involved in proposed FEC algorithm**Step 1:**The original video frames are encoded by the H.264/AVC CABAC entropy coder before transmission to offer compression, encryption, and FEC.**Step 2:**Sign bits of MVDs and TC are selected from the residual data obtained from the entropy coder after compression, which are then extracted from the bitstream to apply H.264 syntax-based selective encryption using 128-bit key XORed with the selected syntax elements.**Step 3:**The encrypted bitstream of video data is encoded through our FEC algorithm at the sending device, which is then sent to the destination device through the noisy transmission channel simulated by the GE model.**Step 4:**The generating function of the FEC encoder divides the encrypted bitstream into *d*-bit data blocks, called datawords, and adds *r* = *c* − *d* parity bits to generate *c*-bits codewords, where *r*, *c*, and *d* represent parity bits, codewords, and datawords, respectively.**Step 5:**The crypto-encoded bitstreams of the codewords are transmitted over the noisy channel, which is employed using the Gilbert–Elliott model.**Step 6:**The checking function of the FEC decoder identifies the valid codewords after receiving the erroneous data at the receiving end. If the codeword is valid, its corresponding dataword is extracted. If the codeword is invalid, the algorithm tries to identify the location of the error by adding the position of the incorrect parity bit and flips the bit at that position. After correcting the possible error, the dataword is extracted from the codeword.**Step 7:**Ciphered MVD and TC is XORed using the symmetric key for decryption with the decoder. Decrypted blocks are then combined to regenerate the original blocks of the videos.

The pseudocode of the *generating function* performed at the sending end consists of the following steps listed in Algorithm 2.
**Algorithm 2** Pseudocode of *generating function* of proposed FEC performed at senderINPUT: Video FramesOUTPUT: H.264 encoded bitstream1:Split the video bit stream into blocks of size *d*2:**for** each block:3:  Generate codewords using bitstream, data block size d, and codeword length *c*
4:  Calculate number if parity bits by *r = c* − *d*5:  **for** *x* = 0 to *r* − 16:    Insert parity bit *r_x_* at 2*^x^* position checks each alternate data bit and skips *x* data bit in the block to maintain even parity7:  
**end for**
8:**end for**

Algorithm 3 lists the steps performed at the receiving end by the *checking function* implemented by the decoder for error detection and correction.
**Algorithm 3** Pseudocode of *checking function* of proposed FEC framework performed at receiving end for error recoveryINPUT: H.264 encoded erroneous bitstreamOUTPUT: Recovered decoded video frames1:Receive the codewords2:**if** the codeword matches any valid codeword3:  Extract codewords by removing parity bits *r_x_* from position 2*^x^* (where *x* = 0, 1, 2, …)4:  Decode the dataword to obtain original video frames5:**else**6:  Detect error7:  **for** each parity bit *r_x_* positioned at 2*^x^* (where *x* = 0, 1, 2, …)8:    Checks each alternate data bit and skips *x* data bit in the block to maintain even parity, i.e., number of 1s should be even, including the parity bit.
    **if**
*r_x_* = expected-parity-bit **then**
    No error has occurred up to that parity bit9:    **else if**
*r_x_* ! = expected parity bit (does not maintain even parity) **then**
10:    Error has occurred11:    Add bit positions of all incorrect parity bits to obtain the position of error bit *e*12:    
**if e = 0 then**
13:       
**set e = 1**
14:    
**else**
15:       
**set e = 0**
16:    
**end if**
17:     
**end if**
18:  
**end for**
19:**end if**

**Figure 5:**
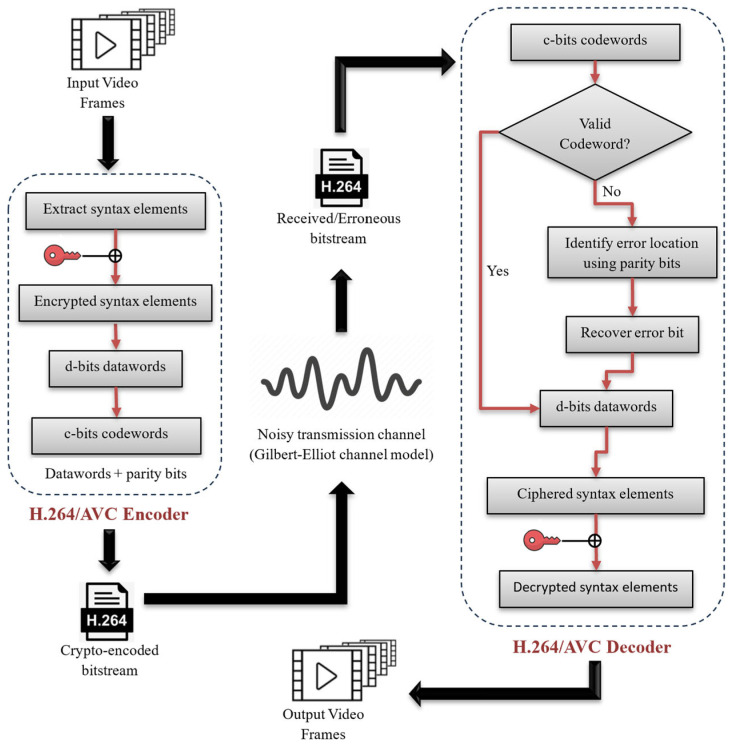
Proposed forward error correction framework for video quality enhancement.

The proposed methodology for the enhancement of video quality using H.264/AVC syntax-based selective encryption and the FEC mechanism is shown in Figure 5.

The state-of-the-art error correction techniques, including machine learning and deep learning approaches, offer high performance potential but exhibit higher computational overhead and require large training and validation sets. By contrast, our proposed FEC mechanism has a computational complexity of *O* (*n*) for encoding and *O* (*n* + *r*) for decoding, where *n* is the number of encoded bits and r is the number of redundant bits. This linear complexity makes our framework more efficient and straightforward to implement, without requiring extensive training data or complex model training. A comparative analysis of the computational complexities of these techniques is given in Table 1.

## 4. Results

To demonstrate our proposed framework, the H.264/AVC encoder was used to simulate an error-prone channel model by inserting errors at multiple points. The videos were first compressed by the H.264/AVC encoder, offering lossless compression to overcome bandwidth limitations. Then, selective encryption of syntax elements (sign bits of MVD and TC) and the proposed FEC algorithm were implemented in H.264/AVC’s CABAC entropy coder during the encoding process. The decoding process was simulated using the H.264/AVC decoder, which decoded and corrected bit errors from the erroneous videos. The implementations were carried out on an HP Spectre x360 Intel Core i7 Processor with 16 GB RAM and a 64-bit operating system. Joint Scalable Video Model (JSVM) 9.19.14 was integrated in Visual Studio 2022 using C++ programming language to deploy the proposed method. As the aim of our work was to enhance the objective visual quality, the proposed scheme was applied on several test video sequences with different features, such as varying amounts of color pixels, texture, objects, and motion vectors. These results provided better visual quality when compared with the results obtained without using the FEC mechanism.

The proposed method was not designed for fixed video resolution and can be applied to videos with different resolutions. In our work, we compared the results on video sequences of two different video resolutions. The results were evaluated on Common Intermediate Format (CIF) (352 × 288) resolution on test video sequences MOBILE and FOOTBALL. For high-definition (HD) (1280 × 720) resolution, the results were assessed using VIDYO1 and FOUR PEOPLE video sequences. These test video sequences are publicly accessible in Derf’s collection. The frame rate was set to 30 fps, GOP size was 16, and subsampling was 4:2:0. H.264/AVC was used to encode and decode the CIF and HD test video sequences. The quality of the video sequences was evaluated through PSNR and PSNR_611_ quality assessment metrics.

Objective video quality can be measured using different evaluation parameters. Depending on the availability of the original video for the comparison, the evaluation methods are categorized as FR, RR, and NR assessment methods, as already described in Section 1. We used the FR technique to evaluate the video quality after recovery from errors by calculating the peak signal-to-noise ratio (PSNR) values. It is the ratio between the original video signal and the signal after passing through a processing scheme. Let y be the number of bits per frame and (2*^y^* − 1)^2^ represents the range of values that a pixel can take, the PSNR is calculated as follows:

(12) $$PSNR=10\log_{10}\frac{{\left(2^{y}-1\right)}^{2}}{MSE}$$

PNSR is the most promising predictor used for evaluating video quality and it is dependent on the mean square error (MSE). MSE specifies the amount of similarity between the original video and the encoded/impaired video [45]. A smaller MSE value means there is less distortion in the processed video, resulting in a higher PSNR value. Therefore, a higher PSNR value means that the video has less distortion and better visual quality.

**Figure 6:**
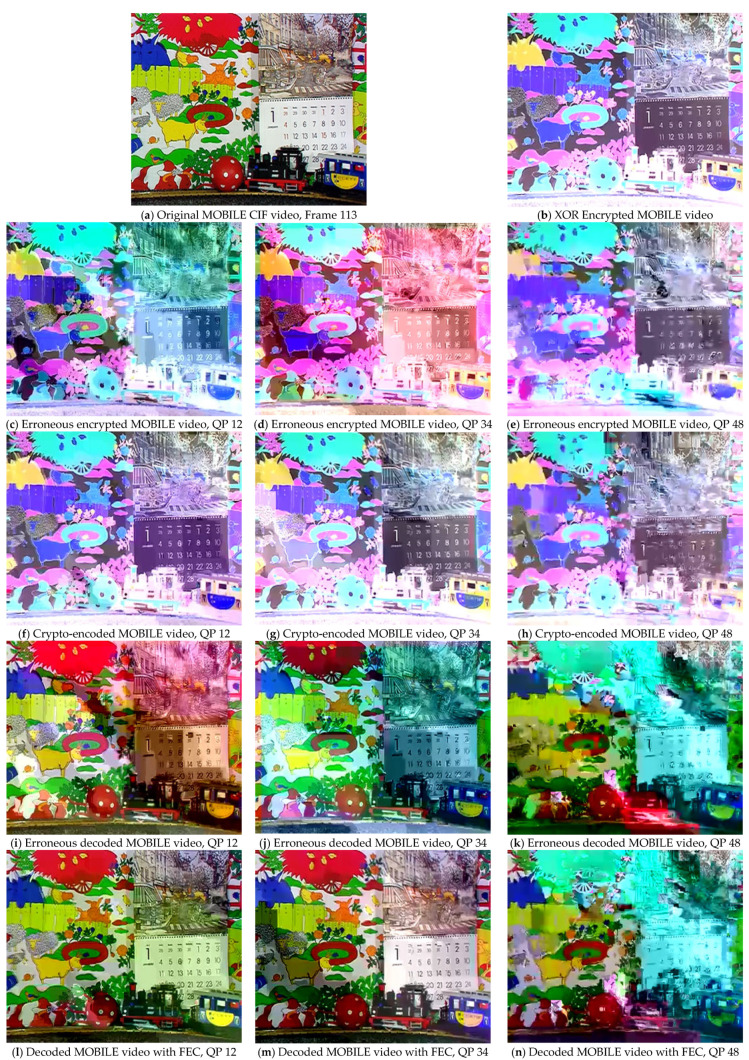
Comparative evaluation of the proposed FEC framework on test sequence MOBILE at QP 12, 34, and 48 in CIF resolution with and without applying the proposed FEC framework.

**Figure 7:**
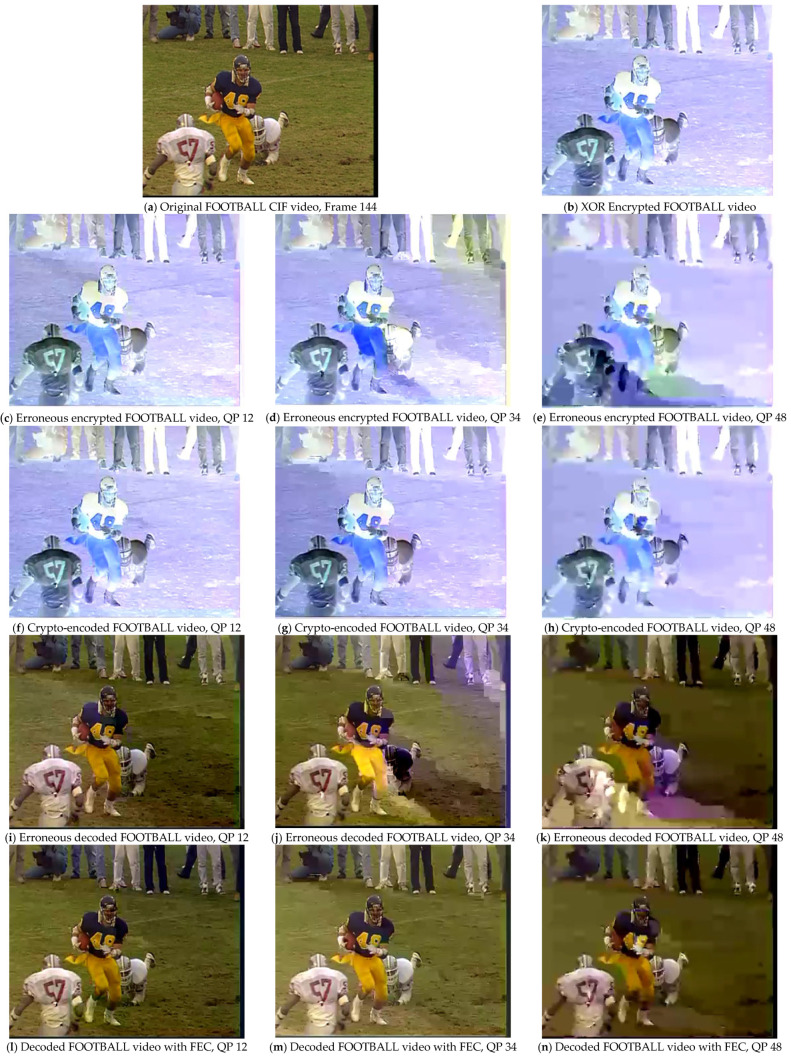
Comparative evaluation of the proposed FEC framework on test sequence FOOTBALL at QP 12, 34 and 48 in CIF resolution with and without applying the proposed FEC framework.

**Figure 8:**
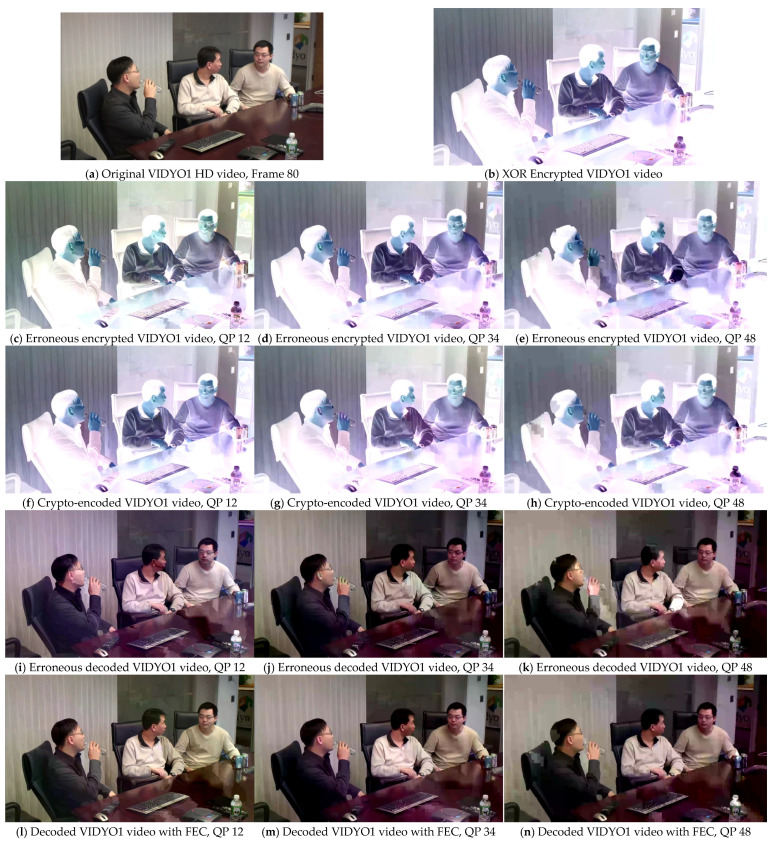
Comparative effect of the proposed FEC on the perceptual quality of test sequence VIDYO1 in HD resolution at QP 12, 34, and 48. (**a**,**b**) show the original and XOR encrypted video frames, respectively; (**c**–**e**) show the erroneous video frames before decryption; (**f**–**h**) show the crypto-encoded video frames; (**i**–**k**) show the video frames decoded without using FEC; and (**l**–**n**) show the video frames after error correction using the proposed FEC technique.

**Figure 9:**
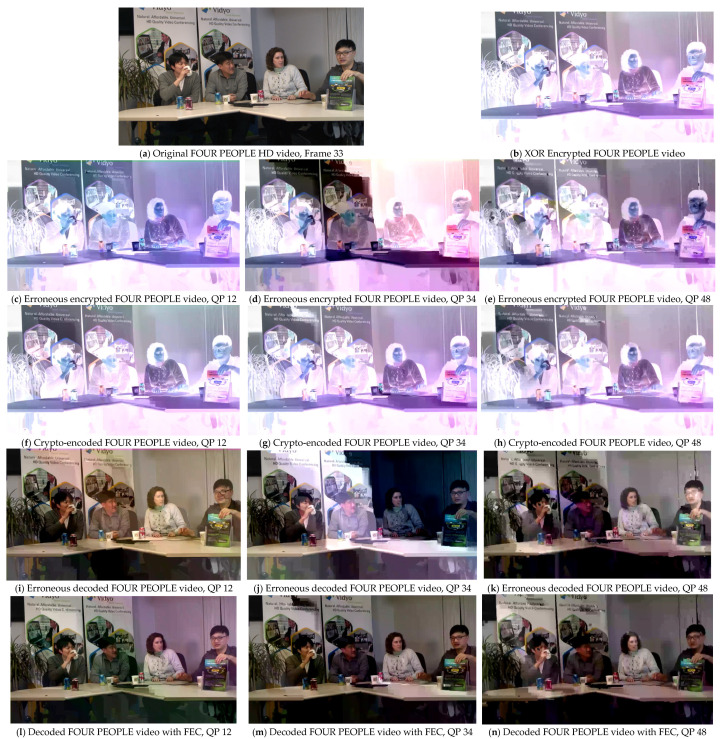
Comparative effect of the proposed FEC on the perceptual quality of test sequence FOUR PEOPLE in HD resolution at QP 12, 34, and 48. (**a**,**b**) show the original and XOR encrypted video frames, respectively; (**c**–**e**) show the erroneous video frames before decryption; (**f**–**h**) show the crypto-encoded video frames; (**i**–**k**) show the video frames decoded without using FEC; and (**l**–**n**) show the video frames after error correction using the proposed FEC technique.

An advanced alternative to calculate the correlation of the perceived video quality introduced during the development of the HEVC coding standard is given below:

(13) $${PSNR}_{611}=(6{PSNR}_{y}+{PSNR}_{u}+{PSNR}_{v})/8$$

where ${PSNR}_{y}$ represents the luminance, whereas ${PSNR}_{u}$ and ${PSNR}_{v}$ refer to blue and red chrominance, respectively. ${PSNR}_{611}$ provides a combined score for luminance and chrominance assessment and relates better to subjective video quality as compared to classical PSNR [24,25]. When there is less distortion or noise, the PSNR_611_ value will be higher, indicating that the quality of the processed video is nearly close to that of the original video. The results were evaluated at three different QP values (12, 34, and 48) to observe the effect of the FEC method through conventional PSNR and advanced PSNR_611_ evaluation metrics on the video sequences. The comparative results with and without the proposed FEC are presented in Figure 6, Figure 7, Figure 8 and Figure 9. The results were compared for video sequences that were encoded without the proposed scheme, crypto-encoded, and decoded without incorporating FEC to analyze the efficiency of the presented scheme. Figure 6a, Figure 7a, Figure 8a and Figure 9a show the original video frame, Figure 6b, Figure 7b, Figure 8b and Figure 9b show the XOR encrypted frame, Figure 6c–e, Figure 7c–e, Figure 8c–e and Figure 9c–e show the encrypted video frames affected due to errors that occurred during transmission before decryption, Figure 6f–h, Figure 7f–h, Figure 8f–h and Figure 9f–h show the crypto-encoded encrypted frames, Figure 6i–k, Figure 7i–k, Figure 8i–k and Figure 9i–k show the video frames decrypted without FEC, and Figure 6l–n, Figure 7l–n, Figure 8l–n and Figure 9l–n show the recovered video frames decoded by the H.264/AVC decoder.

It was observed that the PNSR values in all Y, U, and V components increased in the recovered videos after being decoded using our technique as compared to the PSNR values of the decoded videos affected by the noisy channel. Similarly, the PSNR_611_ values of the erroneous and recovered decoded video frames also exhibited a significant increase in the overall perceptual quality of the video. The PSNR and PSNR_611_ values at different QP values of erroneous, crypto-encoded, decoded without the FEC method, and recovered using FEC test video sequences are summarized in Table 2, Table 3, Table 4 and Table 5.

**Figure 10:**
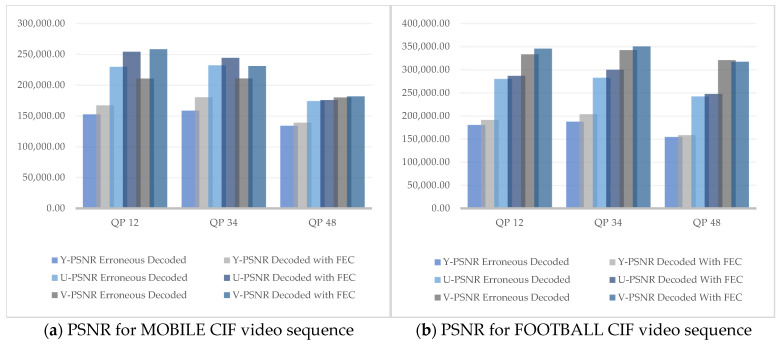

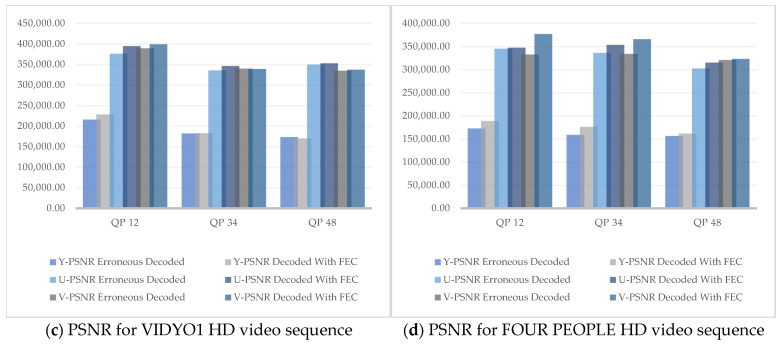
Comparison of Y, U, and V components of PSNR value on MOBILE CIF video sequence.

It was observed from the results that the visual quality at QP 12 was better in terms of chrominance factors, whereas a clear increase in Y-PSNR (luminance component) was observed at QP 34. However, at QP 48 there was a minor increase in visual quality. This is because QP is inversely proportional to video quality. Thus, by increasing the QP, the video quality decreased. The overall increase in visual quality was best noticed at QP 34, as the human visual system is more sensitive to luminance as compared to chrominance. Figure 10a–d illustrate the graphs of different PSNR values at QP 12, 34, and 48 of both the CIF and HD test video sequences.

**Figure 11:**
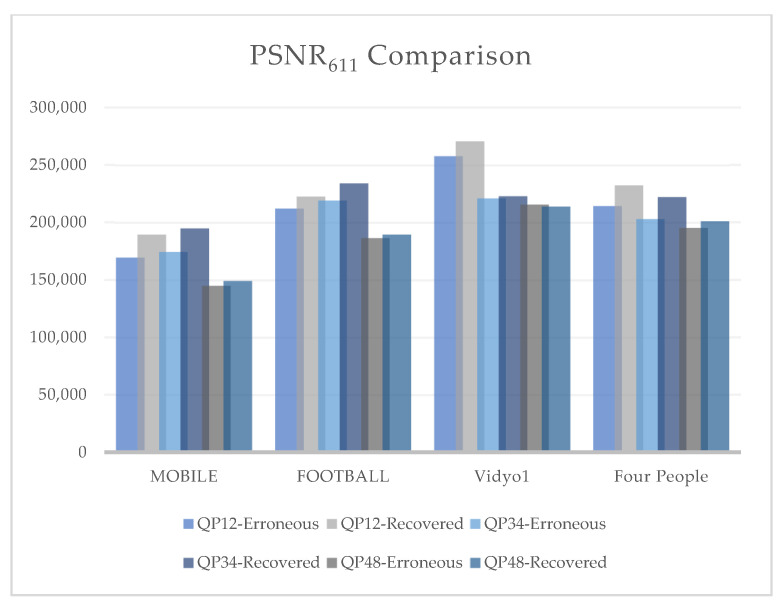
Comparison of PSNR_611_ for MOBILE, FOOTBALL, VIDYO1, and FOUR PEOPLE video sequences at different QP values (12, 34, and 48).

Figure 11 summarizes the effect of PSNR_611_ on the erroneous and recovered videos of our four test sequences. PSNR_611_ evaluates the combined effect of brightness and color components on the perceptual visual quality of the video. The results showed that the combined luma and chroma effect was best noticed at QP 34 for videos with lower resolution and better observed at QP 12 on videos with higher resolution. For the crypto-encoded video frames, the best results were observed at QP 34 irrespective of varying video resolution, as the algorithm’s encryption performance was better at smaller PSNR values. It is clearly observed from the graphs that the proposed FEC algorithm performed best at QP 34. A significant increase in Y-PSNR resulted in enhanced objective visual quality.

**Figure 12:**
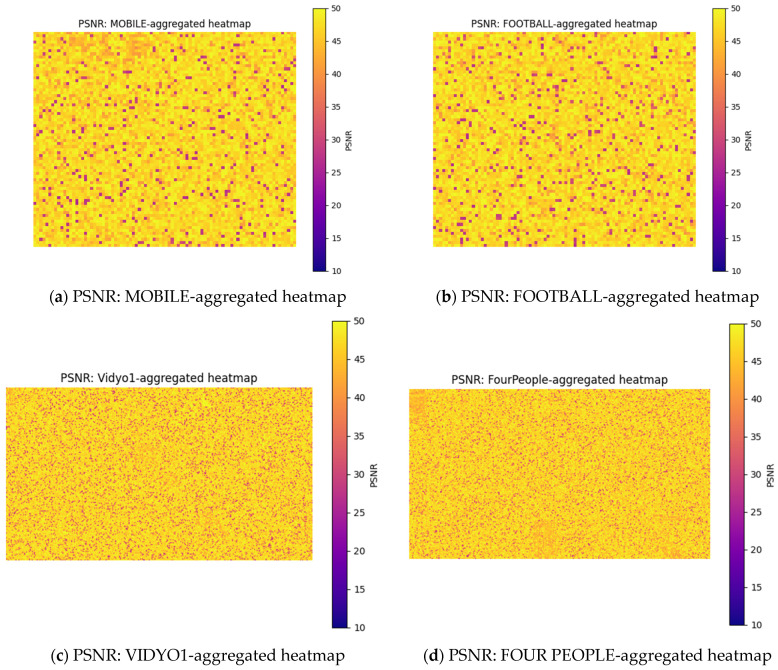
Aggregation of video frames of MOBILE, FOOTBALL, VIDYO1, and FOUR PEOPLE test sequences at QP 34.

Figure 12 illustrates the aggregated heatmaps of these four video sequences at QP 34, where error correction was best observed. Figure 12a shows that the average similarity of the recovered MOBILE video to the original video was around 88–92%. Figure 12b shows that the reconstruction quality of the FOOTBALL video exhibited significant fluctuations due to the high motion and texture complexity inherent in its content. Despite high motion and complex texture, our approach yielded an estimated average similarity of 80–88%, indicating a reasonable level of error recovery. Figure 12c exhibits high reconstruction quality with the consistently yellow heatmap, which indicated high similarity and less distortions, suggesting effective recovery due to lower motion. Its estimated similarity was around 88–95%. Figure 12d presents the heatmap of the FOUR PEOPLE video. The uniform yellow tone with minimal dark areas indicated consistently high PSNR values and an estimated similarity of 87–92%. It suggested better error recovery and reconstruction quality, likely due to limited motion and simple scene complexity. These heatmaps demonstrate the effectiveness of our FEC approach for four videos with varying levels of motion and texture complexity, achieving an average similarity of 90–95%.

## 5. Discussion

In this work, we examined an FEC-based framework to enhance the objective video quality of videos that are affected by noisy transmission channels without compromising confidentiality. The foundation of this work was to devise a simple framework that provides improved visual quality to the viewer by incorporating forward error correction in the encoding process. The video is first compressed to accommodate bandwidth limitations due to its large size and then is selectively encrypted for privacy preservation and protection against unauthorized access. FEC is deployed on the encrypted bitstream during the encoding process, offering a joint crypto-encoding framework. It is observed in the results presented in the previous section that the proposed method can recover the errors and sufficiently supports quality enhancement.

The comparison of the recovered videos with the original videos was evaluated through the PSNR values of the Y, U, and V components of the videos to examine the results in terms of luminance and chrominance factors. PSNR_611_ was used to calculate the collective effect of luma and color components in both erroneous and recovered videos. The results suggested that the PNSR values in each component increased in the FEC decoded videos as compared to the PSNR values of the videos decoded without FEC. The Y component controls the luminance (brightness) and the other two components, U and V, are used to represent chrominance (color). Since the human eye is more sensitive to brightness as compared to color, the results suggested a significant improvement in the Y component. The smaller PSNR values of the encrypted videos showed the better performance of the encryption algorithm. The increased PSNR values meant that the recovered videos had reduced amounts of distortion and the visual quality was restored after decoding with the FEC method.

Moreover, it was observed that the application of this approach was not confined to a specific video resolution, as it provided improved outcomes on both CIF and HD test video sequences. The effect of PSNR also varied at different QPs on the test video sequences. Objective visual quality at QP 12 provided better outcomes in terms of color components, whereas a significant increase in luminance was observed at QP 34. As larger QP values preserve less detail in the quantization process, QP 48 provided a minor increase in the perceptual quality of the video. Therefore, it was clearly observed that increasing the QP provided less improvement in video quality as compared to smaller QP values. In summary, the results suggest that it is possible to employ our technique to achieve better objective video quality, avoiding the need for retransmission and complex computational cost.

There is a potential trade-off between error correction capability and bandwidth expansion, which depends on the number of redundant bits added to generate codewords using data bits. In the  (c,d) encoding scheme, *r* bits are added to *d* data bits to form a codeword having *c* bits. In our (15, 11) encoding scheme, the bandwidth expansion was ((c−d)/d)×100 = ((15 − 11)/11) × 100 = 36.36%. If we further reduce the block size, such as in a (7, 4) encoding scheme, the number of redundant bits will be increased in the original bitstream of data, resulting in a bandwidth expansion of 75%. By contrast, a larger block size such as (255, 247) will incorporate a smaller number of redundant bits in the original bitstream, resulting in lower bandwidth expansion but at the cost of reduced error correction efficiency, specifically, the bandwidth expansion in this case will be 3.24%. Although this approach provides flexibility to adjust the redundancy level according to the available resources, its limitation lies in the fact that choosing a large block size will not be as efficient if the channel is highly noisy and has larger errors. This trade-off between error correction capability and bandwidth expansion necessitates a careful balance between these competing factors, particularly in channels prone to burst errors.

The presented work uses a block coding FEC mechanism to enhance the visual quality of videos by recovering transmission errors. Methods for error recovery using convolution codes can be designed in the future. The proposed algorithm assists in preserving the integrity and confidentiality of transmitted videos by incorporating H.264 syntax-based encryption with the proposed FEC framework and can be tested in the future with other advanced encryption standards to evaluate its impact on encrypted videos. The performance of this work can be further examined on the basis of other quality assessment metrics. Compression efficiency and speed in H.264/AVC has reached the point where it can no longer be further enhanced. Therefore, several new video codecs have emerged to meet the increasing demands of multimedia technology, such as H.265, HEVC/H.265, AV1, and VVC. The effectiveness of this approach can further be evaluated on higher video resolutions (2K, 4K, 8K, etc.) [46]. As our work has been implemented using the H.264/AVC CABAC entropy coder, it can be implemented in modern coding standard H.265 and HEVC for future multimedia transmission, which provides twice the compression as the H.264 coding standard [47,48]. The data reduction efficiency in H.265 is much higher in compressing videos and makes transmission of ultra-high-definition 4K or 8K videos easier [49].

To provide better compression efficiency, reduce complexity, and improve scalability, scalable video coding (SVC) techniques, such as H.264/SVC and VP9, can be optimized to enable efficient video transmission over heterogeneous networks [50]. It can be expanded to a scalable coding environment where the number of parity bits added is dependent on scalable bitstreams to offer variable quality demands for devices with varying resolutions. The cross-layer design and optimization of error correction mechanisms can be explored, where the application layer’s FEC strategy is informed by and coordinates with error correction mechanisms at lower layers. This would potentially lead to more efficient use of resources and improved overall system performance.

## 6. Conclusions

We have presented and implemented an FEC-based framework for H.264/AVC compressed video bitstream. The proposed FEC integrates H.264 syntax element-based selective encryption that improves the quality of videos that have been affected by various types of channel errors during transmission and provides protection against unauthorized access. It is a block coding FEC method that corrects the error bit by finding its location within a macroblock of H.264 encoded video bitstream. It has been tested on multiple test video sequences of CIF and HD resolution at different QP values. QP is the quality of perception and it is reciprocal to the video quality. The results are presented at three different QP values (12, 34, and 48). The effectiveness of the proposed FEC method was analyzed by evaluating the PSNR and PSNR_611_ values of selectively encrypted, noisy encrypted, crypto-encoded, decoded without FEC, and recovered with FEC videos. These results exhibited a significant improvement in the PSNR values of each component (Y, U, and V) and PSNR_611_ values of the videos using the proposed FEC mechanism. The performance of our FEC algorithm was best observed at QP 34. Our method can correct up to 90–95% errors from a 10–15% affected erroneous video by correctly guessing the error bits and reversing those bits to recover the error. It is a simple method that reduces the computational complexity of the codec as it does not involve complex mathematical operations. It combines the encryption process with error correction to avoid the additional computation cost. Moreover, it does not require any backchannel for retransmitting the complete data in case of error occurrence, avoiding transmission delay and making it suitable for real-time applications where low latency is crucial. Therefore, it can be used practically to preserve the objective quality of transmitted videos that has been compromised during transmission due to noisy channels.

## Figures and Tables

**Table 1:** Comparative analysis of our proposed method with these existing techniques.

Technique	Encoding Complexity	Decoding Complexity	Remarks
LDPC [19]	O(n)	$O(n^{2})$	Longer codes increase computational complexity; higher decoding complexity; iterative decoding overhead; matrix sparsity vs. performance trade-off; non-deterministic execution time.
Polar Codes [19]	O(N log N)	O(N log N)	Recursive nature can lead to increased latency and memory requirement; relatively higher computational overhead; implementation complexity for real-time video.
RL-AFEC based on RS codes [15]	O(k·(n−k))	$O(k^{3})$	Efficient for small to moderate k; decoding can be intensive; reinforcement learning would add additional complexity.
QC-LDPC lattice codes [20]	O(e·(n−k)·k)	O(N·(dv · iterations))	Optimized encoding; decoding depends on iterations for convergence; potential security risks may arise from implementation flaws
LSTM based Adaptive FEC [21]	$O(k\cdot n\cdot T\cdot (h\cdot d+h^{2})$	$O(T\cdot (h\cdot d+h^{2})$	High computational and data requirements; complexity of LSTM training; latency that can impact real-time video transmission; suitable for complex patterns.
Proposed FEC	O(n)	O(n+r) or O(n)	Linear complexity; predictable performance; no training required; implementation simplicity; suitable for applications where low latency is critical.

**Table 2:** PSNR values for MOBILE video sequence at QP 12, 34, and 48.

MOBILE Sequence (300 Encoded Frames)	QP	PSNR			PSNR_611_
		Y	U	V	
Selectively Encrypted		6.8953	12.5929	13.5121	84.346
Erroneous encrypted	12	7.4265	12.3083	12.9860	8.7317
	34	7.1810	12.8498	13.0363	8.6216
	48	7.2603	12.4300	13.4313	8.6779
Crypto-encoded	12	7.1660	12.3031	13.3674	8.5834
	34	7.0807	12.1838	13.1670	8.4794
	48	7.1066	12.6136	13.5531	8.6008
Erroneous decoded	12	15.2537	22.9716	21.0694	16.9454
	34	15.8538	23.2130	21.0794	17.4269
	48	13.4094	17.4188	18.0050	14.4851
Decoded with FEC	12	16.7156	25.4193	25.8394	18.9441
	34	18.0556	24.4310	23.0945	19.4824
	48	13.9166	17.5792	18.1795	14.9073

**Table 3:** PSNR results for FOOTBALL video sequence at QP 12, 34, and 48.

FOOTBALL Sequence (260 Encoded Frames)	QP	PSNR			PSNR_611_
		Y	U	V	
Selectively Encrypted		8.6731	13.2727	21.6415	10.8691
Erroneous encrypted	12	8.9489	13.1808	21.8988	11.0967
	34	9.1727	13.3928	21.8384	11.2835
	48	9.0079	14.0011	21.7918	11.2301
Crypto-encoded	12	8.8705	13.2917	21.7341	11.0311
	34	8.9766	13.2443	21.6809	11.0981
	48	8.9004	13.7884	21.5507	11.0927
Erroneous decoded	12	18.0576	28.0026	33.3415	21.2113
	34	18.7813	28.2622	34.2367	21.8984
	48	15.4578	24.2213	32.0710	18.6299
Decoded with FEC	12	19.1280	28.6815	34.5732	22.2529
	34	20.3536	29.9943	35.0606	23.3971
	48	15.8493	24.7632	31.7200	18.9474

**Table 4:** PSNR results for VIDYO1 video sequence at QP 12, 34, and 48.

VIDYO1 Sequence (300 Encoded Frames)	QP	PSNR			PSNR_611_
		Y	U	V	
Selectively Encrypted		5.5501	22.0752	26.7047	10.2601
Erroneous encrypted	12	5.8465	22.2183	26.7082	10.5007
	34	5.9092	22.5080	27.0491	10.6266
	48	5.9062	22.4481	26.9202	10.6007
Crypto-encoded	12	5.8363	22.0415	26.7087	10.4710
	34	5.7507	22.2094	26.9203	10.4543
	48	5.7008	22.3731	26.8432	10.4277
Erroneous decoded	12	21.5816	37.6253	38.9612	25.7596
	34	18.1969	33.5504	33.9977	22.0912
	48	17.3286	34.9941	33.4677	21.5542
Decoded with FEC	12	22.8362	39.4581	39.9339	27.0512
	34	18.2792	34.6328	33.9010	22.2762
	48	17.0078	35.2746	33.7222	21.3805

**Table 5:** PSNR results for FOUR PEOPLE video sequence at QP 12, 34, and 48.

FOUR PEOPLE Sequence (300 Encoded Frames)	QP	PSNR			PSNR_611_
		Y	U	V	
Selectively Encrypted		5.4580	21.0508	25.4978	9.9121
Erroneous encrypted	12	5.9046	21.1821	24.7575	10.1709
	34	6.0077	21.0520	24.8736	10.2465
	48	6.1065	21.4245	25.5034	10.4459
Crypto-encoded	12	5.8462	21.2459	25.5886	10.2390
	34	5.8005	20.9894	25.4370	10.1537
	48	5.6469	21.1603	25.9035	10.1182
Erroneous decoded	12	17.2712	34.5400	33.2581	21.4282
	34	15.8858	33.6038	33.4139	20.2916
	48	15.6434	30.2399	32.0758	19.5221
Decoded with FEC	12	18.8894	34.7405	37.7054	23.2228
	34	17.6168	35.3420	36.5710	22.2018
	48	16.1693	31.5220	32.3041	20.1053

## Data Availability

Data are available upon request from the authors.

## Abbreviations

The following abbreviations are used in this manuscript:

AES	Advanced Encryption Standard
AES-CTR	Advanced Encryption Standard Counter Mode
ARQ	Automatic Repeat Request
AVC	Advanced Video Coding
CABAC	Context-Adaptive Binary Arithmetic Coding
CAVLC	Context-Adaptive Variable-Length Coding
CIF	Common Intermediate Format
CFB	Cipher Feedback Mode
CU	Coding Unit
DCT	Discrete Cosine Transform
EC	Error Concealment
ECC	Error Correcting Codes
ER	Error Resilience
FEC	Forward Error Correction
FHD	Full High Definition
FR	Full Reference
GE	Gilbert–Elliott
GOP	Group of Pictures
HD	High Definition
HEVC	High Efficiency Video Coding
InterPM	Inter-Prediction Mode
IPM	Intra-Prediction Mode
JECC	Joint Encryption and Channel Coding
JENC	Joint Encryption and Network Coding
JESC	Joint Encryption and Source Coding
JSVM	Joint Scalable Video Model
LSTM	Long-Short Term Memory
MSE	Mean Square Error
MV	Motion Vector
MVD	Motion Vector Difference
NR	No Reference
OSI	Open System Interconnection
PSNR	Peak Signal-to-Noise Ratio
QC-LDPC	Quasi-Cyclic Low-Density Parity Check
QP	Quality Perception
QoE	Quality of Experience
QoS	Quality of Service
RC	Residual Coefficient
RL-AFEC	Reinforcement Learning—Adaptive Forward Error Correction
RR	Reduced Reference
RS	Reed–Solomon
SNR	Signal-to-Noise Ratio
SSIM	Structural Similarity Index Method
ST-RRED	Spatio-Temporal Reduced Reference Entropic Differencing
SVC	Scalable Video Coding
TC	Transform Coefficient
TU	Transform Unit
UHD	Ultra-High Definition
VMAF	Video Multimethod Assessment Fusion
VVC	Versatile Video Coding
